# Reassessing the effects of continuous positive airway pressure (CPAP) on arterial stiffness and peripheral blood derived CD34+ progenitor cells in subjects with sleep apnea

**DOI:** 10.1186/s13287-019-1251-8

**Published:** 2019-05-21

**Authors:** Cleyton C. Domingues, Fiona J. Dore, Alexander Cho, Neeki Ahmadi, Yana Kropotova, Nabanita Kundu, Naji Younes, Vivek Jain, Sabyasachi Sen

**Affiliations:** 1 Department of Medicine, School of Medicine and Health Science, The George Washington University, 2300 I Street NW, Washington, DC, 20037 USA; 2 The GW Medical Faculty Associates, Washington, DC, USA; 3 The GW Milken Institute of Public Health, Washington, DC, USA

**Keywords:** Obstructive sleep apnea, Continuous positive airway pressure, Endothelial progenitor cells, Arterial stiffness

## Abstract

**Background:**

Obstructive sleep apnea (OSA) is an independent risk factor for cardiovascular diseases (CVD) and vascular health. Peripheral blood-derived CD34+ progenitor cells have been used as biomarker for CVD risk and may play a similar role in OSA and CVD risk assessment. Although there are some controversial results in the literature, OSA patients may have a reduction in the number and function of CD34+ cells. The damages promoted by OSA in CD34+ cells may lead to an increase in endothelial oxidative stress and endothelial inflammation which may lead to a reduced endothelial repair capacity. In this study, we explored the effect of continuous positive airway pressure (CPAP) on peripheral blood-derived CD34+ cells and arterial stiffness (another predictor of endothelial health and CVD risk) in OSA patients.

**Methods and results:**

Nine overweight and obese subjects without prediabetes or diabetes were recruited. Eight out of nine subjects had moderate to severe degree of OSA. CD34+ cells were isolated from peripheral blood. Number and function of these cells were monitored before and after 3 months of treatment with CPAP. No significant changes were observed in the number of CD34+ cells, CFU-Hill’s colony formation unit (CFU) count or migratory response to the chemotactic factor SDF-1a after CPAP use. However, CXCR4 mRNA expression significantly increased by 2.2-fold indicating that CPAP may have a positive effect on SDF1a receptor (CXCR4), thereby improving migration of CD34+ cells mediated by SDF1a after the 3 month period. Interestingly, in clinical arena our results showed a reduction of pulse wave velocity (an established parameter of arterial stiffness) following CPAP therapy.

**Conclusions:**

Our findings suggest that 3-month CPAP intervention does not show statistical significant increase in CD34+ cell number and function, in mostly moderate to severe OSA subjects; however, it did demonstrate a positive trend. CPAP therapy, did help improve arterial stiffness parameter.

**Electronic supplementary material:**

The online version of this article (10.1186/s13287-019-1251-8) contains supplementary material, which is available to authorized users.

## Background

Obstructive sleep apnea (OSA) is a disorder that affects children and adults and is characterized by repetitive apneas, causing hypoxemia, and unintended arousals from sleep [1]. The prevalence of OSA in adults in the general population can be as high as 38% with a higher incidence in men [2]. In the past decades, the number of people diagnosed with sleep apnea has increased; for instance, a recent study reported that among US male veterans, the prevalence of sleep apnea increased from 3.7 to 8.1% between 2005 and 2014 [3]. Untreated OSA is an independent risk factor for hypertension, myocardial ischemia, and stroke [4]. The mechanisms between OSA and cardiovascular diseases (CVD) are not well understood; however, data suggest that OSA affects the endothelium adversely which might be contributing to the pathophysiology behind cardiovascular disease from OSA. This endothelial dysfunction is an early marker for vascular abnormalities preceding clinically overt CVD. OSA patients who are otherwise free of clinically apparent cardiovascular comorbidities have increased endothelial oxidative stress, inflammation, and reduced endothelial repair capability, thereby strongly suggesting that OSA independently impairs endothelial function [5]. The proposed mechanisms are believed to be due to intermittent hypoxia (similar to an ischemic-reperfusion injury), sleep fragmentation and deprivation (shown to have a 50% decline in endothelial dependent vasodilation), and genetics [6, 7].

Although there have been many different markers of inflammation used to look at endothelial dysfunction, cell-based biomarker such as hematopoietic progenitor cells CD34+ cells to assess endothelial function and treat cardiovascular diseases [8–12] has been widely used in various clinical scenarios [10]. Peripheral blood- and bone marrow-derived hematopoietic CD34+ cells are deemed to be premature or progenitor cells where CD34 is a progenitor marker. These cells have been shown to give rise to capillary formation or tube formation in the presence of appropriate growth factors or environment [11, 13] and develop into mature cobble stone appearing endothelial cells [11]. These CD34+ cells have also been designated as endothelial progenitor cells (EPCs) [14]. Circulating EPCs are intrinsic to vascular repair and regeneration [13] and have been used as a regenerative tool in myocardial ischemia and diabetic wound healing [13–15]. Endothelial dysfunction with associated inflammation may be a consequence of increased oxidative stress in a setting of OSA. Therefore, this pro-oxidative stress condition causes CD34+ cell dysfunction and senescence [16, 17]. Most studies have described a reduction in EPCs in that population, whereas others have shown an increase or no changes in the number of EPCs in OSA [18]. Despite the fact that many studies on EPCs in OSA have been reported, results are still controversial as definition of EPCs without a cell surface marker can be confusing [10]. In OSA patients, a reduction in circulating CD34+ cells can occur, where CD34+ cells may succumb to apoptosis secondary to increased level of reactive oxygen species (ROS) presence. CD34+ cell apoptosis has been noted in other high ROS states, such as hyperglycemia [11]. Increased cellular apoptosis is associated with inflammation, oxidative stress, and increased sympathetic activation with concomitant reduction in EPC mobilization in OSA [18]. An intervention is crucial to reduce or reverse cardiovascular diseases. Considering that continuous positive airway pressure (CPAP) may revert that scenario, in this study, we explored the effects of CPAP treatment on CD34+ cells impairment and arterial stiffness in a particular cohort of OSA patients. Approximately 90% of these patients had apnea hypopnea index (AHI) ranging from 26 to 70 (which is moderate to severe).

## Methods

### Participants

Nine adults with OSA (as defined by apnea-hypopnea index AHI ≥ 5) who had chosen continuous positive airway pressure (CPAP) for their OSA treatment were enrolled. Subjects were over 18 years of age and were diagnosed by an in-house polysomnography (see inclusion/exclusion criteria in Additional file 1). This study consisted of a single site: The George Washington University Medical Faculty Associates. Informed consent was obtained from all patients prior to engaging in study activities. The study was approved by The George Washington University Institutional Review Board (IRB no. 011616).

### Study design

The study consisted of two visits, 3 months or 12 weeks apart. Basic demographics such as age, gender, race/ethnicity and BMI were collected, as well as data from their polysomnography test. At baseline, considered here as visit 1 (prior to initiation of CPAP), we performed a venous blood draw to investigate patients’ CD34+ cell number, function, and gene expression (see below). Routine blood laboratory tests were also performed. All outcome measures were repeated at visit 2, 3 months after treatment with CPAP.

### Polysomnography (PSG) set up

The diagnostic PSG and CPAP titration PSG were conducting using a Respironics Alice 6 LDXN Sleep Diagnostic System (Philips Respironics, Murrsyville, PA) at the Center for Sleep Disorders at the Medical Faculty Associates at The George Washington University. A standard set of clinical measurements was acquired during the PSG, including electrocardiogram, electroencephalogram, oxyhemoglobin saturation, and respiratory airflow. Registered polysomnographic technologists manually scored the sleep studies and identified sleep stages, apneic and hypopneic events, and arousals using standard AASM criteria [19]. Hypopneas and apneas were defined per AASM criteria.

### Arterial stiffness measurements

Arterial stiffness was assessed through pulse wave analysis (PWA) and pulse wave velocity (PWV) using the AtCor SphygmoCor CP system. PWA was obtained from the radial artery while the subjects were seated at rest. An average of two measurements was obtained per visit with an operator index above 80. PWA measures include augmentation index (AI), augmentation index adjusted for a heart rate of 75 (AI-75), augmentation pressure (AP), and both systolic and diastolic blood pressures measured both centrally and peripherally. PWV measures the velocity of the pulse as it moves from a proximal artery (carotid) to a distal artery (femoral). For the PWV test, the subject was lying supine on an exam table. An average of two tests was obtained per visit. Higher values of AP, AI, Ai-75, and PWV are correlated to higher levels of arterial stiffness.

### Evaluation of endothelial progenitor cells

Peripheral blood samples (approximately 60 ml) were drawn from patients and diluted in phosphate buffered saline (1:1). Mononuclear cells (MNCs) were then isolated from whole blood using a Ficoll density centrifugation method. MNCs were counted, and an aliquot was used for CFU-Hill colony formation assay following the manufacturer’s instructions (#05900, Stem Cell Technologies, Vancouver, BC, Canada). At day 5, colony forming units (CFU) were counted. To isolate EPCs (CD34+), MNCs were magnetically sorted through a column after cells were stained with CD34 microbeads antibody (Miltenyi Biotec GmbH, Bergisch Gladbach, Germany). An aliquot of CD34+ cells were then stained with trypan blue and counted using an Auto Cellometer Mini (Nexcelom Bioscience, Lawrence, MA). CD34+ gene expression analysis was performed by quantitative reverse transcriptase polymerase chain reaction (qRT-PCR). CD34+ total mRNA was extracted and purified using the RNeasy mini kit (Qiagen). mRNA was then converted into cDNA by using the high capacity cDNA reverse transcription kit (Applied Biosystems). Gene expressions of samples obtained at both visits were assessed by a CFX96 real-time qPCR system (Bio-Rad) using TaqMan Universal Master Mix II (Applied Biosystems) and inventoried probes. The gene expression analysis included endothelial function (VEGFA, KDR, NOS3), cell chemotaxis (SDF1a, CXCR4), and endothelial lineage cell surface marker (PECAM1). The expression of individual gene was normalized to either housekeeping 18S or GAPDH and calculated by using the 2^−ΔΔCt^ method considering the difference in cycle threshold between visit 2 and baseline (visit 1). The migratory capacity of CD34+ cells were evaluated using the CytoSelect 24-well Cell Migration Assay kit (Cell Biolabs, Inc., San Diego, CA). Cells were suspended in serum-free media and seeded at 100,000 cells per insert. Migration of the cells through a 3-μm polycarbonate membrane to the wells containing serum-free media (control) and chemoattractant SDF-1α (10 or 100 ng/mL) was assessed after cells were kept overnight in a CO_2_ incubator at 37 °C. Migratory cells were dissociated from the membrane and subsequently lysed and quantified by fluorescence (480 nm/530 nm) using CyQuant GR dye (Cell Biolabs, Inc., San Diego, CA). The fluorescence ratios between cells exposed to the chemotactic factor and cells exposed to chemoattractant-free media (control) along the visits were used to analyze the migratory capacity of the cells.

### Statistical analysis

This is a pilot study for proof of concept. Our objective was to determine if 12 weeks of CPAP therapy is sufficient time to positively affect OSA parameters and parameters of CD34+ cells in moderate to severe middle-aged OSA subjects.

Baseline characteristics are described using means and standard deviations for quantitative variables and count and percentage for categorical variables. Tables and figures that compare quantitative variables between visits 1 and 2 show means and standard deviations for each visit and a *p* value from a paired Student *t* test. All *p* values are nominal, with no adjustments for multiple comparisons. Where effect sizes are reported, they are calculated as Cohen’s *d* statistics for paired observations.

## Results

The demographic characteristics of the subjects enrolled in this study according to their severity of sleep apnea as well as the CPAP usage are described in Table 1. Patient ages ranged from 28 to 64 years with an average of 41.3 years. Their BMIs ranged from 25.3 to 35.9 with an average of 27.2, and their baseline AHIs ranged from 14.5 to 69.8. AHI and oxygen desaturation levels are used to indicate the severity of obstructive sleep apnea.

**Table 1:** Baseline demographic and CPAP usage

	All
*N*	9
Gender	
F	2 (22.2%)
M	7 (77.8%)
Age at baseline	41.3 ± 11.9
BMI	27.2 ± 3.4
AHI	32.6 ± 15.3
Severity of sleep apnea	
Mild (5 ≤ AHI < 15 per hour)	1 (11.1%)
Moderate (15 ≤ AHI < 30 per hour)	4 (44.4%)
Severe (AHI ≥ 30 per hour)	4 (44.4%)
Mean % of oxygen desaturation	95.2 ± 1.2
Severity of oxygen desaturation	
Mild	2 (22.2%)
Moderate	5 (55.6%)
Severe	1 (11.1%)
Unlisted	1 (11.1%)
CPAP usage	
Total days	86 ± 3.1
Usage days	63.1 ± 19.9
Usage days %	0.7 ± 0.2
Usage days > 4 h	44.2 ± 28.5
Usage days > 4 h %	0.5 ± 0.3
Usage hours/usage day	4.9 ± 2

Blood biochemistry results showed no significant difference between visits for all the parameters except for blood urea nitrogen (BUN) that was reduced by approximately 13% (nominal *p* < 0.05) (see in Additional file 1: Table S1).

### Analysis of CD34+ cells

**Fig. 1:**
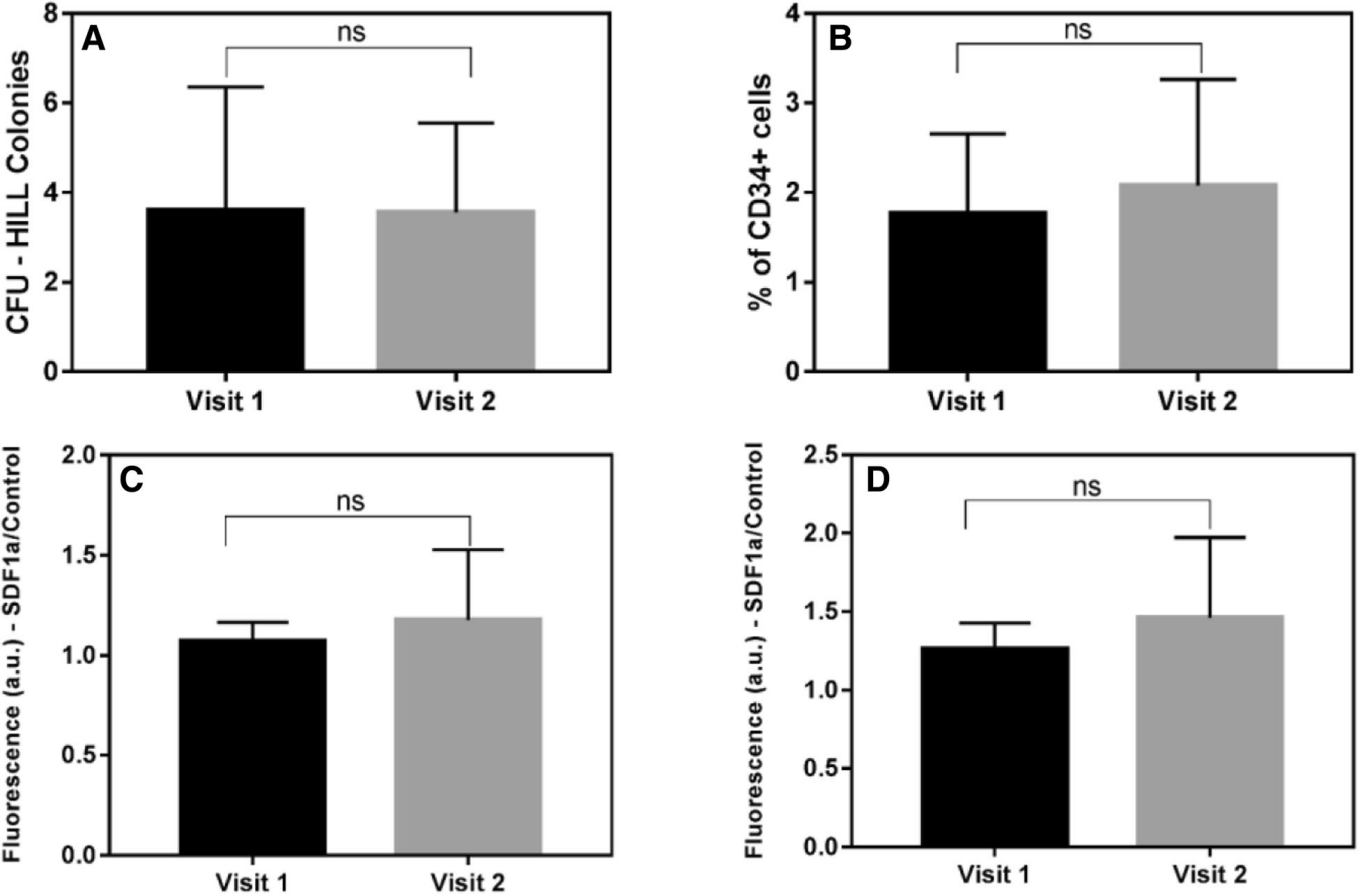
**a** Number of CFU-Hill’s colonies formed by sorted MNCs. **b** Percentage of CD34+ cells isolated from MNCs. 1.2-fold increase in visit 2. **c**, **d** Migration of CD34+ cells in response to SDF-1a (10 and 100 ng/mL); results are expressed as fluorescence ratio between cells exposed to the chemotactic factor and cells exposed to media that did not contain SDF1a as a chemotactic factor (control). 1.1 and 1.2-fold, increase in visit 2, respectively ns = not significant

The CFU-Hill’s colony formation units derived from the MNCs population did not improve by CPAP treatment (Fig. 1a). Although the percentage of CD34+ cells (isolated from MNCs by a magnetic column) did not statistically change (Fig. 1b), the percentage obtained at visit 2 was higher (1.2-fold).

A similar improvement trend was observed following CPAP for the migratory response of CD34+ cells to the chemotactic factor SDF-1a at both concentrations 10 and 100 ng/mL (1.1 and 1.2-fold, respectively; Fig. 1c, d).

**Fig. 2:**
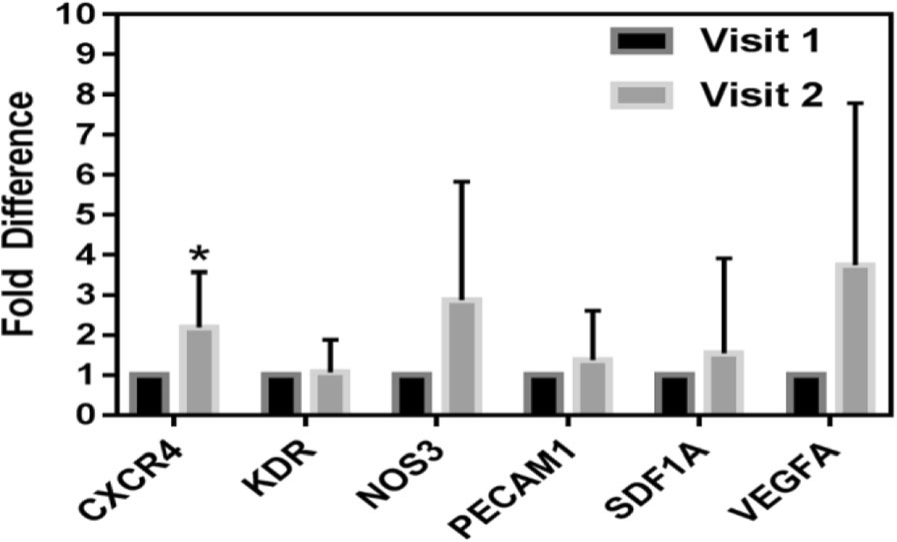
Effect of CPAP on gene expression of vascular genes in CD34+ cells. Results are relative to visit 1 (control); *p < 0.05, paired Student’s *t* test. CD34+ gene expression analysis showed a statistical significant upregulation (2.2-fold, *p* < 0.05) of CXCR4 mRNA expression with increase in expression of NOS3 and VEGF-A

CD34+ gene expression analysis showed an upregulation (2.2-fold, *p* < 0.05) of CXCR4 mRNA expression (Fig. 2).

These results taken together may indicate that CPAP may have a positive effect on number and mobilization of CD34+ cells mediated via the receptor CXCR4, a known receptor for the chemotactic factor SDF1. A 12-week intervention was clearly not enough to show a statistical change in moderate to severe OSA; however, a positive trend was noted.

Subsequently, this process should facilitate homing of endothelial progenitor cells and thus leading to repair of the damaged and inflamed endothelium in sleep apnea subjects. Moreover, like SDF1a—another well-known cell chemotactic factor—VEGFA also showed upregulation with CPAP use with increase in expression of NOS3 a gene that encodes the endothelial nitric oxide synthase enzyme (Fig. 2). A well-established mature endothelium marker so-called PECAM1 also showed a trend of upregulation. No significant changes were found for VEGF Receptor-2 or kinase domain receptor (KDR).

### Arterial stiffness

Table 2 shows arterial stiffness and radial blood pressure measures across the two visits. Although we did not observe a statistical significant change between the two visits neither for systolic nor for diastolic blood pressure, a reduction for pulse wave velocity (PWV) was found to be close to statistical significance (*p* = 0.063). Since arterial stiffness is an indicator of CVD risk [12, 20], a reduction of PWV results indicate a possible reduction in arterial stiffness following CPAP therapy indicating positive cardiovascular outcomes. However, other parameters of arterial stiffness such as augmentation pressure and augmentation index demonstrated no statically significant difference.

**Table 2:** Arterial stiffness parameters in patients subjected to 3 months of CPAP

	Visit 1	Visit 2	*p* value	Effect size
Pulse wave velocity	8.60 ± 1.99	7.48 ± 2.37	0.063	0.72
Systolic blood pressure (radial)	126.56 ± 13.13	122.00 ± 11.37	0.232	0.43
Diastolic blood pressure (radial)	82.06 ± 7.84	79.44 ± 9.51	0.379	0.31
Augmentation index-75	13.17 ± 12.60	13.69 ± 14.48	0.846	0.07
Augmentation pressure	4.56 ± 5.43	5.15 ± 5.20	0.622	0.15

## Discussion

Endothelial dysfunction has been described as an early maker for atherosclerosis ([21] and references therein). There have been several markers used to try and measure endothelial damage including inflammatory markers, cytokines, and EPCs [22, 23]. EPCs have been studied as a marker of endothelial function that may be able to predict future endothelial health and inflammation in OSA with conflicting results [18, 24]. The controversial data in the literature may be partially caused by a small number of patients that has been enrolled in those studies [18, 25], and another factor could be differences in the cohort that were studied such as degree of OSA which has varying ranges of AHI. Definition of EPCs in various studies also could be a confounding factor with different harvesting and isolation techniques from MNC population [10]. Although there are mixed results regarding whether EPCs number increase with treatment of CPAP, there seems to be a consensus that EPC levels are lower in OSA patients in comparison to normal individuals [18]. Our study shows real-life results of CPAP treatment in a nine consecutive OSA patients enrolled with a moderate (*n* = 4) to severe (*n* = 4) degree of AHI score, which had on an average more than 4 h of CPAP usage per day. Our results showed a trend for an increase of the number of CD34+ cells from subjects using CPAP. Additionally, we also observed a trend for an improvement of the cell (CD34+) function such as migratory response to SDF1a as a chemotactic factor. Interestingly, Jelic et al [5, 26] have previously shown that the number of circulating EPCs either normalized or increased after CPAP treatment in newly diagnosed OSA patients (AHI ≥ 5). Therefore, it seems that the effect of CPAP on the number of circulating EPCs depends also on the severity and duration of the disease. We acknowledge that the number of subjects monitored in this study is small and the results presented here should be interpreted with caution and needs to be repeated with a larger cohort. Another methodological factor that can cause misinterpretation of the results is how EPCs are assessed [10, 18]. Here, we sorted CD34+ cells using microbeads antibody and magnetic columns [12]. Although we did not assess EPCs using different marker combinations (i.e., CD34, CD133, KDR), we believe that CD34+ cells still represent EPCs [27]. Moreover, those common markers are not specific [28], and cells expressing CD34+CD133+KDR+ may also not be true EPCs [29].

Despite the limitations of this study, our results corroborate with previous findings showing a positive effect of CPAP on EPCs. However, we would like to stress our results specifically correlate to CD34+ cells in moderate to severe OSA. Compared to literature, our data indicates that the improvement of EPCs (CD34+) number and function may be reduced in a population having a more severe condition of the apnea syndrome.

Since SDF1-CXCR4 interaction plays a key role on EPCs, our hypothesis is that CPAP favors CD34+ cells homing, mobilization, and differentiation via upregulation of the receptor CXCR4 in addition to VEGFA and endothelial nitric oxide. Therefore, the use of CPAP can facilitate endothelium repair process to slow down the progress of endothelial dysfunction in OSA. In fact, it is already known that VEGF expression is increased in the plasma of OSA patients [18, 30]. This might be an adaptive mechanism in order to prevent CVD. Our results showed that CPAP can also increase the mRNA expression of VEGFA in CD34+ cells but no changes were found for KDR mRNA expression. Plasma level of SDF1 in relation to EPCs can be changed depending on the condition (normal vs ischemia acute phase). There is currently a lack of data regarding the effect CPAP on SDF1 plasma levels.

Arterial stiffness (AS) is a noninvasive marker used to evaluate hypertension, arterial stiffness and vascular health in general, and increased stiffness indicates poor vessel contractility. It is associated with an increased risk of cardiovascular complications such as myocardial infarction and cerebrovascular accident [20, 31]. Moreover, AS is also a reliable marker for endothelial cell dysfunction (ECD) and endothelial inflammation [32]. An increase in AS, measured by pulse wave analysis (PWA) and pulse wave velocity (PWV), is associated with an increase in cardiac afterload which can lead to cardiac complications ([33] and references therein). AS could play an integral role in helping to categorize the vascular inflammatory effects of OSA. We noted a trend in improvement or reduction of pulse wave velocity indicating possible improved ECD. Studies on AS have shown more reliable reproducibility with PWV rather than PWA [10, 20].

We also noted a reduction (13%) of BUN with CPAP use; however, no change in creatinine was noted. It is likely that CPAP use improves renal function, but this finding needs to be verified with a larger cohort study.

## Conclusions

In our study, we demonstrated that CPAP has potential benefits in mostly moderate to severe OSA patients with 12 weeks of intervention but the improvement in the number and functionality of EPCs (CD34+) as well as in AS is limited according to the severity of the sleep apnea degree. Our results indicate that CPAP can potentially improve endothelial dysfunction as evidenced by CD34+ cell data and AS data. Further studies with larger cohorts are needed for a better understanding of the effects and mechanisms of action of CPAP on CD34+ cells. The increased gene expression of CXCR4 in CD34+ cells and a trend towards increase in mRNA expression for related genes that control homing-in property of endothelial progenitor cells such as VEGFA and NOS3 support the hypothesis that CPAP can improve the functionality of CD34+ cells but the severity of the sleep apnea should be considered. Our results suggest that CXCR4 gene upregulation of CD34+ cells is involved in the molecular mechanism underlying the improvement of vascular health in OSA.

## Additional file

Additional file 1: Inclusion and exclusion criteria. **Table S1.** Blood biochemistry before and after CPAP treatment. (DOCX 18 kb)

## Abbreviations

AI: Augmentation index
AP: Augmentation pressure
BMI: Body mass index
CFU: Colony forming units
CPAP: Continuous positive airway pressure apnea-hypopnea index AHI
EPCs: Endothelial progenitor cells
MNCs: Mononuclear cells
OSA: Obstructive sleep apnea
PSG: Polysomnography
PWA: Pulse wave analysis
PWV: Pulse wave velocity
VEGFA: Vascular endothelial growth factor A
KDR: Kinase insert domain receptor
NOS3: Nitric oxide synthase 3
SDF1a: Stromal cell derived factor 1 a
CXCR4: C-X-C Motif chemokine receptor 4
PECAM1: Platelet and Endothelial cell adhesion molecule 1
18S: 18 Svedberg ribosomal RNA
GAPDH: Glyceraldehyde-3-phosphate dehydrogenase
*CXCL12a SDF1a*: C-X-C motif chemokine 12 stromal cell-derived factor 1
BUN: Blood urea nitrogen
CVD: Cardiovascular disease
