# Supplementary material for: Reassessing the effects of continuous positive airway pressure (CPAP) on arterial stiffness and peripheral blood derived CD34+ progenitor cells in subjects with sleep apnea
Source: Stem Cell Res Ther. 2019 May 21;10:147. doi: 10.1186/s13287-019-1251-8 (PMC6530134; doi:10.1186/s13287-019-1251-8)
Supplement: Supplementary file 1 — Inclusion and exclusion criteria. Table S1. Blood biochemistry before and after CPAP treatment. (DOCX 18 kb) [file 13287_2019_1251_MOESM1_ESM.docx]

**SUPPLEMENTAL MATERIALS**

**INCLUSION AND EXCLUSION CRITERIA**

**Inclusion Criteria:**

- CPAP naive male and female adults (> 18 years of age) with diagnosis of obstructive sleep apnea as measured by in-laboratory polysomnography (sleep study). They must have chosen to undergo CPAP treatment for their OSA.

**Exclusion Criteria:**

- Prior CPAP treatment.
- Subjects not willing to or otherwise unable to use CPAP for treatment of OSA.
- Presence of other sleep disorders.
- Diagnosis of diabetes mellitus Type 1 and 2 (known to independently affect EPC function).
- Previous cardiovascular or cerebrovascular disease (known to independently affect EPC function).
- Smokers (Current or within the past 5 years, known to independently affect EPC function).
- Recently began using or dose change of statins.
- Current, chronic use of anti-inflammatory drugs (known to independently affect EPC function).
- Pregnant or breastfeeding women.
- Women of child bearing age (WOCBA) not willing to or unable to use an acceptable method to avoid pregnancy for the entire study period.
- Prisoners or subjects who are involuntarily incarcerated.
- Subjects who are compulsorily detained for treatment of either a psychiatric or physical (e.g., infectious disease) illness.
- Subjects unable to give consent because of language barrier, or any other reason.

**Table 1S: BLOOD BIOCHEMISTRY BEFORE AND AFTER CPAP TREATMENT**

|  | **Visit 1** | **Visit 2** | **p-value** |
| --- | --- | --- | --- |
| **Glucose**  Fasting (mg/dL)  HbA1c (%) | 86.2 ± 8.9  5.3 ± 0.2 | 88.9 ± 7.2  5.5 ± 0.2 | 0.343  0.150 |
| **Lipid panel** (mg/dl)  Total cholesterol  Triglycerides  HDL  VLDL  LDL  LDL/HDL  Cholesterol/HDL | 186 ± 38.1  114.2 ± 55.8  52.3 ± 14.7  22.9 ± 11.3  111.3 ± 29.7  2.2 ± 0.7  3.7 ± 0.8 | 190 ± 42.8  142.6 ± 73  52.6 ± 17.5  28.4 ± 14.5  109 ± 34.8  2.2 ± 0.8  3.8 ± 1 | 0.325  0.483  0.216  0.510  0.283  0.604  0.906 |
| **Electrolytes** (mmol/L)  Sodium  Potassium  Chloride  Carbon dioxide  Calcium | 139.3 ± 6.1  6.1 ± 4.9  103.7 ± 13.4  23.1 ± 7.3  9.6 ± 0.3 | 141.7 ± 1.3  4.4 ± 0.3  100.6 ± 2.2  23 ± 1.3  9.4 ± 0.3 | 0.433  0.330  0.411  0.984  0.052 |
| **Liver function** (units)  ALPL  AST  ALT  Total protein (mg)  Serum albumin  Total bilirubin  Total globulin  A/G | 60.2 ± 26.4  34.8 ± 48.1  29.8 ± 16.2  8.9 ± 5.3  5.2 ± 1.7  0.8 ± 0.8  3.2 ± 1.8  2.2 ± 1 | 71.1 ± 14.2  19.7 ± 3.9  21.6 ± 8.7  7 ± 0.3  4.5 ± 0.2  0.3 ± 0.1  2.5 ± 0.3  1.9 ± 0.2 | 0.356  0.619  0.279  0.355  0.348  0.226  0.321  0.414 |
| **Kidney function**  BUN  Serum creatinine  BUN/creatinine  eGFR non-African American  eGFR African American | 15 ± 2.1  0.9 ± 0.2  27.6 ± 30.3  91.3 ± 31  104.8 ± 37.3 | 13.1 ± 2.5  0.8 ± 0.2  16.7 ± 4.5  102.7 ± 11.1  118.6 ± 13 | 0.033*  0.119  0.353  0.183  0.188 |
